# An equity-lens analysis of policies on child health in the Democratic Republic of the Congo

**DOI:** 10.1186/s41182-025-00857-8

**Published:** 2025-11-22

**Authors:** Etienne Mwokozi Bwira, Paulin Beya Mutombo, Théophane Kekemb Bukele, John Kebela Kamwina, Dosithée Ngo-Bebe

**Affiliations:** 1 Department of Health Policy and Management, School of Public Health, University of Kinshasa, Kinshasa, Democratic Republic of the Congo; 2 Department of Nutrition, School of Public Health, University of Kinshasa, Kinshasa, Democratic Republic of the Congo; 3 Department of Epidemiology and Biostatistics, School of Public Health, University of Kinshasa, Kinshasa, Democratic Republic of the Congo

**Keywords:** Health equity, Policy analysis, EquiFrame, Child health, Democratic Republic of the Congo

## Abstract

**Background:**

Despite policy commitments to equitable healthcare, the Democratic Republic of the Congo (DRC) continues to experience significant disparities in child health outcomes. While national health policies formally express support for equity in access to health services, there is limited evidence on the extent to which they incorporate the core concepts (CCs) of equity. This study assessed the extent to which equity is embedded in child health policies.

**Methods:**

The EquiFrame framework was used to analyse five child health policy documents selected based on their recency, public availability, and strategic relevance. In this study, 16 equity-related CCs were employed to evaluate both the extent of their coverage and the quality of the commitment demonstrated across the selected policy documents. Each document was analysed and ranked as low, moderate, or high in addressing equity-related CCs.

**Results:**

The National Strategic Plan to Combat Malaria is the only policy document that achieved a high equity rating. The remaining documents were ranked as moderate. Overall, 44% of equity concepts were consistently included across all reviewed documents. While Access, Prevention, Quality, Capacity Building, Integration, and Participation were the most frequently addressed equity-related CCs, critical concepts such as Non-discrimination, Cultural Responsiveness, and Individualized Services were completely omitted. In most cases, the policy frameworks lacked the operational detail, clearly defined measurable actions and robust monitoring mechanisms required to achieve a meaningful impact.

**Conclusion:**

This study revealed significant gaps in addressing equity in child health policies in the DRC. Future policies should systematically incorporate all equity-related CCs, accompanied by clear, measurable actions and robust monitoring frameworks. Strengthening these components is essential to advance equitable access to child health services and ensure that all children, regardless of background or circumstance, can achieve their full health potential.

## Introduction

The World Health Organization (WHO) defines equity as “the absence of unfair, avoidable, or remediable differences among groups of people, whether defined socially, economically, demographically, geographically, or by other dimensions of inequality” [1]. Achieving health equity means every individual has the opportunity to reach their full potential for health and well-being [2], recognizing health as a basic human right [3, 4].

As an ethical principle closely tied to human rights, equity provides a strong foundation for translating the right to health into actionable policies and practices [4]. To achieve equitable health outcomes, it is essential to integrate key human rights principles, such as access, quality, non-discrimination, participation, and accountability, into health policies [5]. These principles promote equity by removing structural barriers that disproportionately affect marginalized groups, including women, children, people with disabilities, and those living in poverty [6, 7]. Evidence shows that applying these principles in health policy and practice leads to measurable improvements in healthcare access, quality, and outcomes, especially among disadvantaged populations [8, 9]. For example, promoting non-discrimination and cultural responsiveness enhances inclusiveness, while upholding rights to information, privacy, and participation fosters trust and encourages service utilization [10, 11]. On the other hand, the lack of clear equity guidance in health policies in many countries has contributed to persistent inequalities, such as higher child morbidity in low-income households, limited access to essential healthcare, and inequitable resource distribution [12–14].

Achieving equitable health outcomes is a central goal of global health initiatives, including Universal Health Coverage (UHC) and Sustainable Development Goal (SDG) 3 [15]. These global health efforts have led to measurable progress, notably in reducing maternal, infant, and child mortality [16]. Although progress has been made, health inequities remain widespread within health systems, particularly in low- and middle-income countries (LMICs) [17, 18]. In Africa, many health policies have not adequately addressed inequities due to top-down policy development processes, which often exclude community participation and fail to align with local needs [19, 20]. Furthermore, entrenched political and economic interests frequently distort resource allocation, widening the gap between policy intentions and community realities and perpetuating health disparities [19].

In the Democratic Republic of the Congo (DRC), health equity has been a long-standing priority, particularly in the aftermath of the political and socio-economic crises of the 1990s. Guided by the principles of "Primary Health Care" established at the 1978 Alma-Ata Conference, the DRC adopted a pro-poor health approach supported by both the government and development partners [21]. The National Health Policy of 2001 reaffirmed the government’s commitment to equitable healthcare, emphasizing ethical service provision and fair resource distribution [21]. This commitment is further enshrined in Article 47 of the 2006 Constitution, which guarantees the right to health as a fundamental right of all Congolese citizens [22]. To operationalize this vision, the Ministry of Public Health launched the Health System Strengthening Strategy in 2006, implemented through consecutive five-year National Health Development Plans (NHDPs) [23]. The 2011–2015 NHDP, the first in this series, focused on the WHO’s six health system building blocks and supported the pursuit of Millennium Development Goals [24]. The subsequent 2016–2020 NHDP aimed to expand access to quality, equitable, and affordable health services, advancing progress toward UHC and the health-related SDGs, with a particular focus on community care and child survival [25]. Building on the mid-term evaluation of the 2016–2020 plan, the 2019–2022 NHDP emphasized high-impact maternal, newborn, and child health interventions, including emergency obstetric and neonatal care, integrated management of childhood illnesses, expanded immunization, and family planning [26]. This plan was complemented by the 2019–2022 Integrated Strategic Plan for Reproductive, Maternal, Newborn, Child, and Adolescent Health and Nutrition, aligning with national priorities and the 2016–2030 UN Global Strategy for Women’s, Children’s, and Adolescents’ Health [27].

These sustained efforts have led to measurable improvements in child health outcomes. Nationally representative surveys show that the under-five mortality rate declined from 148 deaths per 1,000 live births in 2007 [28] to 93 in 2023 [29]. Similarly, the infant mortality rate dropped from 92 to 56 deaths per 1,000 live births over the same period [28, 29]. Despite this progress, the DRC remains among the countries with the highest child mortality rates globally and is not on track to achieve child survival-related SDG targets if current trends continue [30].

Although notable progress has been made in child health at the national level, considerable disparities persist across socio-economic groups, geographic regions, and between urban and rural areas [31, 32]. For example, marked disparities in full immunization coverage persist, with higher rates among children from the richest households (24%), urban areas (19%), and mothers with higher education (32%) compared to those from the poorest households (5%), rural areas (7%), and mothers with no education (4%) [29].

Although the DRC has adopted policies and plans that express a commitment to equity and the right to health, it remains unclear to what extent these documents support child health-related human rights. In particular, there is a lack of research evaluating how effectively national health policies incorporate human rights and equity dimensions in the context of child health. To address this gap, this study assessed the extent to which equity is embedded in child health policies and identified policy documents that may require revision. To achieve this, we applied EquiFrame, a peer-reviewed framework developed by Mannan et al. [37] to evaluate the inclusion of core human rights principles in health policies, particularly in relation to vulnerable populations. The framework has been widely used to assess policy content across various countries [33–36]. A detailed description of the framework has been described elsewhere [37].

## Methods

This study employed a qualitative descriptive design to conduct a policy content analysis of five key policy documents issued at the national level in the DRC.

### Study setting

The DRC is the largest nation in sub-Saharan Africa, a low-income country with an estimated population of 105.6 million in 2024 [38]. In the past 30 years, the country has faced prolonged armed conflicts, persistent political instability, and complex humanitarian crises. The public health system in the DRC is structured across three tiers: central, provincial (or intermediate), and peripheral (or operational). The central level is responsible for establishing policies, standards, and regulations, while providing technical support, oversight, and monitoring for their implementation at the provincial level [26]. At the central level, the Directorate of Family and Specific Groups is responsible for coordinating the implementation and monitoring of Maternal, Newborn and Child health (MNCH) activities nationwide [27]. Given the complexity of MNCH challenges, a dedicated MNCH Task Force has been established at both the central and provincial levels. This Task Force serves as a platform for collaboration and knowledge exchange between programmes, technical and financial partners, academic institutions, and civil society organizations involved in MNCH. Nevertheless, significant challenges persist, particularly regarding the limited institutional integration of programmes and projects and the fragmented formulation of action plans. Each programme or project involved in MNCH tends to develop its plan in isolation, with minimal coordination or alignment with other existing initiatives in the field [27]. In addition, MNCH providers are unevenly distributed across the country, with marked disparities between provinces and a concentration in urban centres, leaving many rural areas underserved [39].

### Selection of policy and strategic plan documents

**Fig. 1:**
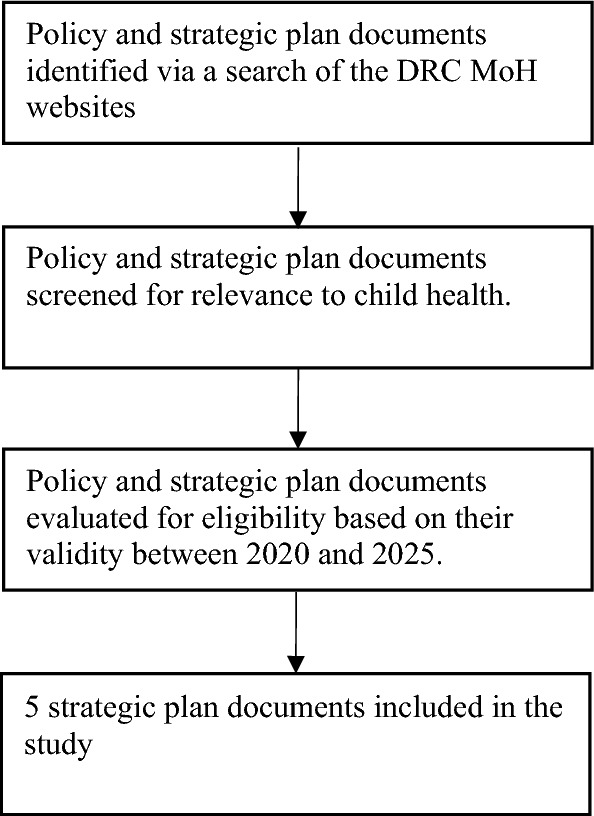
Process of selecting policy documents

This study focused on the most recent publicly available policies and strategic plans relevant to child health. The selected health policy documents met the following eligibility criteria: (i) child health-related or overarching policy documents; (ii) issued or endorsed by the Ministry of Health (MoH); (iii) valid between 2020 and 2025; and (iv) applicable at the national level. We conducted a search for eligible policy documents through the official websites of the DRC MoH. Applying these selection criteria resulted in the inclusion of five policy and strategic plan documents, as presented in Fig. 1. The documents analysed were as follows:

1. Reframed National Health Development Plan (NHDP), 2019–2022 [26];
2. National Strategic Plan to Combat Malaria, 2020–2023 [40];
3. Integrated Strategic Plan for Reproductive, Maternal, Newborn, Child, and Adolescent Health and Nutrition (RMNCAH-NUT), 2019–2022 [27];
4. National Multisectoral Strategic Nutrition Plan, 2023–2030 [41];
5. Comprehensive Multi-Year Plan for Immunization (CMYPI), 2020–2024 [42].

### Data analysis

For this study, the EquiFrame framework [37] guided the content analysis of health policy and strategic plan documents. This policy analysis tool was designed to systematically assess the integration of 21 key human rights concepts and the consideration of 12 vulnerable groups in health policies, aiming to enhance equity in healthcare. According to the authors of EquiFrame, the framework provides an analytical approach that can be adapted to the specific requirements of the analysis. To address specific needs, as well as political, cultural, or contextual interests or constraints, equity-related core concepts (CCs) can be added or removed. This study applied an adapted version of the EquiFrame framework to align the tool with the specific objective of our analysis. Consequently, we reduced the number of CCs from 21 to 16, selecting those most relevant to the context and documents under review. These CCs are presented in Table 1, along with their definitions.

**Table 1:** List of EquiFrame core concepts applied in the policy analysis

No.	Core concept	Definition
1	Access	This assessed the extent to which a policy or plan supported vulnerable groups physical, economic, and information access to health services
2	Accountability	This assessed the extent to which a policy or plan specified to whom, and for what, services providers are accountable
3	Capacity building	This assessed the extent to which a policy or plan supported the capacity building of health workers and of the system that they work in addressing health needs of vulnerable groups
4	Coordination of services	This assessed the extent to which a policy or plan supported assistance of vulnerable groups in accessing services from within a single provider system (interagency) or more than one provider system (intra-agency) or more than one sector (intersectoral)
5	Cultural responsiveness	This assessed the extent to which a policy or plan ensured services responded to the beliefs, values, gender, interpersonal styles, attitudes, cultural, ethnic, or linguistic, aspects of the person
6	Efficiency	This assessed the extent to which a policy or plan supported efficiency by providing a structured way of matching health system resources with service demands to address health needs of vulnerable groups
7	Entitlement	This assessed the extent to which a policy or plan indicated how vulnerable groups may qualify for specific benefits relevant to them
8	Family resource	This assessed the extent to which a policy or plan recognized the value of the family members of vulnerable groups in addressing health needs
9	Individualized services	This assessed the extent to which a policy or plan supported the rights of vulnerable groups to receive individually tailored services to meet their needs and choices
10	Integration	This assessed the extent to which a policy or plan promoted the use of mainstream services by vulnerable groups
11	Non-discrimination	This assessed the extent to which a policy or plan supported the rights of vulnerable groups to have equal opportunity in receiving health care
12	Participation	This assessed the extent to which a policy or plan supported the right of vulnerable groups to participate in the decisions that affect their lives and enhance their empowerment
13	Prevention	This assessed whether the policy or plan supported vulnerable groups in seeking primary, secondary, and tertiary prevention of health conditions
14	Privacy	This assessed the extent to which a policy or plan addressed the need for information regarding vulnerable groups to be kept private and confidential
15	Protection from harm	This assessed the extent to which a policy or plan provided protection for vulnerable groups from harm during their interactions with health and related systems
16	Quality	This assessed the extent to which a policy or plan supported quality services to vulnerable groups through highlighting the need for evidence-based and professionally skilled practice

Adapted from EquiFrame [37]; 16 of the 21 original core concepts were retained

The analysis and scoring method provided by the EquiFrame framework was straightforward and practical. Five key Child health policy documents were evaluated using this framework.

The analysis of the policy documents involved two main steps recommended by the EquiFrame. First, each CC was assigned a score ranging from 0 to 4, reflecting the quality of commitment to the CC within each policy document, as shown in Table 2. Second, three summary indices (CC coverage, CC quality and overall summary ranking) were obtained for each policy document. The details of the calculation and interpretation of these summary indices are provided in Table 2.

**Table 2:** Core concept scoring and summary indices for policy document rating

Document analysis	Description
*Core concept scoring*	
0	Concept not mentioned
1	Concept only mentioned
2	Concept mentioned and explained
3	Specific policy actions identified to address the concept
4	Intention to monitor the concept was expressed
*Summary indices*	
Core concept coverage	The percentage of CCs incorporated into the policy out of the total number of CCs. CC coverage is determined by the formula (*n*/16) × 100, where n is the number of core concepts scoring above 0, and 16 is the total number of CCs."
Core concept quality	The percentage of CCs rated as three or four out of the total number of CCs. CC quality is calculated by dividing the total number of CCs scoring three or four by 16, then multiplying by 100
Overall ranking of policy or plan document	Policies and plans were ranked according to their performance in the CC coverage and quality categories. The ranking criteria were as follows: high = if the policy achieved ≥ 50% on both scores above; moderate = if the policy achieved ≥ 50% on one of two scores above; and low = if the policy achieved < 50% on both scores above

### Quality assurance

To ensure the quality of the analysis, two reviewers independently evaluated each policy document for the inclusion of CCs. Their scores were compared, and any discrepancies were resolved through discussion. If disagreements persisted, consensus was reached in consultation with other research team members. The final scores were then used to calculate the summary indices across all included documents. Moreover, since the selected DRC policy documents were written in French, we used the officially validated and published French version of the EquiFrame Framework manual to avoid potential biases associated with self-translation from English to French [43].

## Results

The analysis assessed five strategic plans related to child health in the DRC via the EquiFrame framework. Table 3 shows the core equity concept scores for the five DRC national child health policy documents, while Table 4 presents the EquiFrame summary indices of coverage, quality, and the overall ranking.

**Table 3:** Core equity concept scores across five national child health policy documents in the DRC

Core concepts	Policy document				
	Reframed NHDP	National Strategic Plan to Combat Malaria	Integrated Strategic Plan for RMNCAH-NUT	National Multisectoral Strategic Nutrition Plan	CMYPI
Access^a^	3	3	4	4	3
Accountability^a^	2	2	1	2	1
Capacity building^a^	3	3	4	3	3
Coordination of services	1	1	0	1	0
Cultural responsiveness^b^	0	0	0	0	0
Efficiency	1	0	1	1	1
Entitlement	3	0	0	3	0
Family resource	0	4	0	4	0
Individualized services^b^	0	0	0	0	0
Integration^a^	1	3	3	1	3
Non-discrimination^b^	0	0	0	0	0
Participation^a^	3	3	3	1	3
Prevention^a^	3	4	3	4	3
Privacy	0	0	0	1	0
Protection from harm	1	3	0	4	0
Quality^a^	3	3	4	4	3

^a^Mentioned across all five policy documents

^b^Not mentioned in any policy document

**Table 4:** Ranking of the equity analysis of policy documents related to child health

Policy document	CC coverage (%)	CC quality (%)	Overall ranking
Reframed NHDP	69	38	Moderate
National strategic plan to combat malaria	63	50	High
Integrated strategic plan for RMNCAH-NUT	50	38	Moderate
National multisectoral strategic nutrition plan	81	44	Moderate
CMYPI	50	38	Moderate

Across all reviewed documents, seven of the 16 equity concepts (44%) were consistently addressed. These concepts included Access, Accountability, Capacity Building, Integration, Participation, Prevention, and Quality. However, the concepts of Cultural Responsiveness, Non-Discrimination and Individualized Services were not mentioned in any of the five policies reviewed. All five policies met EquiFrame’s 50% criterion for CC Coverage. Only the National Strategic Plan to Combat Malaria met the 50% threshold for CC Quality and received a high overall ranking. The remaining four policies, which did not meet this criterion, were classified as moderate. Building on these overall findings, the detailed results for each individual child health policy document are presented below.

### The reframed National Health Development Plan

Eleven of the 16 CCs achieved a score greater than zero in the reframed NHDP, resulting in a CC Coverage score of 69%. The concepts of Cultural Responsiveness, Family Resource, Individualized Services, Non-discrimination, and Privacy were not mentioned in the policy. In terms of CC Quality, six concepts integrated in the policy received a score of 3. This resulted in a CC Quality score of 38% for the policy. The concepts of Access, Capacity Building, Entitlement, Participation, Prevention, and Quality were each mentioned in the policy, accompanied by specific actions designed to support their implementation. The concepts of Coordination of Services, Efficiency, Integration, and Protection from harm were mentioned only, while Accountability was mentioned and explained in the policy. The reframed NHDP scored above 50% on EquiFrame’s summary index of CC coverage, while scoring below 50% on CC Quality. As a result, it received a Moderate Overall Summary Ranking.

### The National Strategic Plan to Combat Malaria

The National Strategic Plan to Combat Malaria incorporated 10 of the 16 CCs, resulting in a CC Coverage score of 63%. The concepts of Cultural Responsiveness, Efficiency, Entitlement, Individualized Services, Non-discrimination, and Privacy were not mentioned in the policy. Regarding CC Quality, the concepts of Access, Capacity building, Integration, Participation, Protection from harm, and Quality each scored 3, indicating that the policy identified specific actions to address these concepts. The concepts of Family Resource and Prevention scored 4, showing that the policy expressed an intention to monitor them. Overall, eight of the 16 CCs received a score of 3 or 4, resulting in a CC Quality score of 50% for the policy. The concept of Coordination of services was only mentioned, whereas Accountability was mentioned and explained. By meeting the 50% threshold for EquiFrame’s Summary Index of CC Quality and exceeding the 50% criterion for Core CC Coverage, the National Strategic Plan to Combat Malaria was assigned a High Overall Summary Ranking.

### The Integrated Strategic Plan for RMNCAH and Nutrition

The Integrated Strategic Plan for RMNCAH and Nutrition addressed eight of the 16 CCs, resulting in a CC Coverage score of 50%. The concepts of Coordination of services, Cultural Responsiveness, Entitlement, Family Resource, Individualized Services, Non-discrimination, Privacy, and Protection from harm were not mentioned in the policy. Six of the mentioned CCs received scores of 3 or 4; accordingly, the policy achieved a CC Quality score of 38%. The concepts of Access, Capacity building, and Quality scored 4, reflecting that the policy expressed an intention to monitor these concepts. Integration, Participation, and Prevention scored 3, indicating that specific policy actions were included in the policy to address them. However, the concepts of Accountability and Efficiency were only mentioned. The policy met the EquiFrame criterion of 50% for CC Coverage but fell below the 50% threshold for CC Quality. The Overall Summary Ranking of the Integrated Strategic Plan for RMNCAH and Nutrition was Moderate.

### The National Multisectoral Strategic Nutrition Plan

The National Multisectoral Strategic Nutrition Plan scored 81% with respect to CC Coverage. The concepts of Cultural Responsiveness, Individualized Services, and Non-discrimination were not mentioned in the policy. With seven concepts scoring 3 or 4, the policy achieved a CC Quality score of 44%. The concepts of Access, Family Resource, Prevention, Protection from Harm, and Quality were mentioned in the policy, with an explicit intention to monitor them. The concepts of Capacity Building and Entitlement were each mentioned alongside specific policy actions aimed at addressing these concepts. The concepts of Coordination of services, Efficiency, Integration, Participation, and Privacy were only mentioned, whereas Accountability was mentioned and explained. While the policy exceeded the EquiFrame criterion of 50% for CC Coverage, it did not reach the 50% threshold for CC Quality. Therefore, the overall summary ranking of the National Multisectoral Strategic Nutrition Plan was Moderate.

### The Comprehensive Multi-Year Plan for Immunization

The CMYPI achieved a CC Coverage score of 50%. The concepts of Coordination of services, Cultural Responsiveness, Entitlement, Family Resource, Individualized Services, Non-discrimination, Privacy, and Protection from harm were not mentioned in the policy. The policy scored 38% for CC Quality. The concepts of Access, Capacity Building, Integration, Participation, Prevention, and Quality were mentioned in connection with specific policy actions designed to address them. The concepts of Accountability and Efficiency were only mentioned in the policy. The policy met the EquiFrame criterion of 50% for CC Coverage but fell below the 50% criterion for CC Quality, resulting in a Moderate Overall Summary Ranking.

## Discussion

This policy analysis examined the extent to which five national strategic plans related to child health in the DRC incorporated equity principles, using an adapted version of the EquiFrame framework. Only the National Strategic Plan to Combat Malaria achieved a high equity ranking, whereas all other policies received a moderate overall rating. These findings align with previous evidence from Ethiopia, where an assessment of MNCH policies similarly revealed that most were ranked as moderate [44]. The limited achievement of high-quality equity integration underscores the urgent need for more structured and explicit strategies to mainstream equity into child health policy in the DRC.

The overall analysis showed that less than half of the core equity concepts were consistently addressed across all five strategic plans. CC coverage at the document level varied substantially, ranging from 50 to 81%. These levels of CC coverage are relatively low compared with findings from previous studies conducted in Sudan [33], Malawi [34], and Ethiopia [44], which reported higher coverage of equity-related core concepts. The limited inclusion of core equity concepts across the policy landscape is particularly concerning in a context such as the DRC, where child health disparities remain widespread [31, 32].

The analysis of equity concept quality revealed even greater weaknesses. Only one of the five policy documents examined met the 50% quality threshold, while the others demonstrated varying degrees of underperformance. In most cases, the intention to monitor core equity concepts was not explicitly expressed across all policy documents examined. Similar findings have been reported in other sub-Saharan African countries [34, 44], where policy reviews also highlighted the absence of dedicated monitoring and evaluation (M&E) frameworks to support the implementation and assessment of equity-related commitments. This pattern suggests a disconnect between policy rhetoric and practical implementation, a challenge well documented in global health policy research [45].

At the concept-specific level, Access, Capacity Building, Participation, Prevention, and Quality emerged as the most consistently represented, with scores between 3 and 4 across all five plans. This suggests a baseline recognition of the importance of reducing barriers to services, strengthening the health workforce, promoting preventive and quality care, and involving communities in programme delivery, areas emphasized in global child health agendas [46]. Although Integration was addressed in all of the reviewed policy documents, it was assigned a score of only 1 in two cases. This reveals gaps in guaranteeing that vulnerable populations can effectively access and benefit from general health and related services.

However, several concepts have been addressed inconsistently or in vague terms. Accountability, while present in all policy documents with scores ranging from one to two, often lacked clearly defined enforcement mechanisms. The absence of robust accountability frameworks is a well-documented barrier to equitable health service delivery and governance [45]. The uneven inclusion of Coordination of services and Entitlement reflects challenges in harmonizing multisectoral efforts and enshrining rights-based approaches in policy, which are critical for comprehensive equity but often difficult to translate into practice [47]. The variability observed in the inclusion of Protection from harm raises concerns about the safeguarding of vulnerable children, a principle emphasized in international child rights frameworks but frequently overlooked in policy implementation [48]. Family Resource, a determinant encompassing socio-economic support and caregiving capacity, shows a fragmented policy approach. While two plans addressed it with specificity, its omission from the others points to a lack of consensus on the role of family involvement in promoting health equity. This gap is particularly concerning given the substantial evidence highlighting the effectiveness and sustainability of community-based interventions that actively engage families [49]. Efficiency, although included in some plans, was superficially treated, indicating a missed opportunity to optimize resource use in the context of constrained health financing, a challenge well recognized in LMICs [50].

The most striking gaps were observed in the complete omission of three out of sixteen core equity concepts. Cultural Responsiveness, Individualized Services, and Non-discrimination were not mentioned in any of the five reviewed documents. The absence of these principles is especially worrisome in the context of the DRC’s rich cultural and social diversity, as it risks further marginalizing already disadvantaged populations, including children from ethnic minorities, displaced communities, and those living in remote or underserved areas [38]. The lack of attention to Non-discrimination is alarming, as it contradicts international norms on the right to health and equitable access to care [51]. Similarly, the near total neglect of Privacy and confidentiality considerations reflects a gap in aligning national policy with internationally accepted standards for patient rights and trust in healthcare services [11].

### Policy recommendations

In light of the equity analysis results presented above, we propose the following recommendations:

Policymakers in the DRC should explicitly integrate the CCs of Non-discrimination, Cultural Responsiveness, and Individualized Services into all child health policies to reduce persistent inequities in access and outcomes. Policies grounded in non-discrimination ensure equitable service availability and prevent biased exclusion, particularly among marginalized populations in rural and conflict-affected areas [52, 53]. Cultural responsiveness can foster trust and adherence by aligning health interventions with sociolinguistic and traditional norms in the DRC’s diverse communities [37, 54]. Individualized services strengthen effectiveness by tailoring care to children with unique vulnerabilities, including those with disabilities, at risk of malnutrition, or with limited family support [55]. Incorporating individualized services into policy frameworks also enhances continuity of care, facilitates the early identification of at-risk children, and supports family-centred decision-making, ultimately contributing to more equitable and effective child health outcomes [56].

Policies should also more robustly address the concepts of Accountability, Entitlement, Coordination of Services, Protection from harm, Privacy, Family Resource, and Efficiency to advance equitable child health outcomes. Strengthening accountability ensures that healthcare providers and institutions are responsible for delivering high-quality and equitable services, helping to reduce gaps in service delivery, treatment availability, and follow-up care for vulnerable children [57]. Reinforcing entitlement guarantees every child’s right to essential health services, improving access for those in rural, impoverished, or humanitarian settings where preventable illness and mortality remain high [53, 58]. Improved coordination among government sectors, non-governmental organizations, civil society, and community groups can reduce duplication, optimize resource use, and ensure integrated, continuous care, particularly in remote and conflict-affected areas [57]. Enhancing protection from harm prevents abuse, unsafe clinical practices, and hazardous environments that undermine caregiver confidence and delay care-seeking [58, 59]. Ensuring privacy builds trust, encourages utilization of services, and supports sensitive care such as HIV interventions [60, 61]. Expanding family resource support, including financial or social assistance, reduces care-related costs and strengthens families’ ability to adhere to treatment and follow-up visits [62, 63]. Improving efficiency ensures that limited resources, including supply chains and frontline health workers, are optimally used, enabling timely and uninterrupted care [47, 64].

Finally, when revising policy documents, policymakers should integrate a robust, equity-focused M&E framework with indicators disaggregated by region, socio-economic status, gender, and other vulnerable groups. Embedding this framework into routine data systems and allocating dedicated resources to programmes targeting underserved populations will enable timely detection of disparities, track progress toward equity goals, strengthen accountability, and ensure that revised policies translate into measurable improvements in child health outcomes [52].

### Strengths and limitations

This study has some strengths that enhance its validity and relevance. It applies an adapted version of the widely used and peer-reviewed EquiFrame analytical framework to systematically assess the extent to which core equity principles are integrated into national child health policies. In addition, this analytic tool can be used not only for retrospective policy analysis but also for evaluating current policies to track progress in addressing equity. By focusing on a largely overlooked area in the DRC, this study helps fill an important knowledge gap.

However, the study has certain limitations. Firstly, the analysis only included five national policy documents related to child health, which may have excluded other relevant sectoral or subnational policies. Consequently, the findings may not fully capture the broader policy environment that shapes child health equity in the DRC. Secondly, the study relied solely on document review, which may limit our understanding of how equity principles are interpreted and applied in practice. Including the perspectives of policymakers, programme managers, and frontline workers could provide deeper insights into their implementation. Finally, some equity concepts may have been implicitly embedded within broader policy objectives without being explicitly stated, which could lead to their presence being underestimated. Future research should therefore broaden its scope to include sectoral and subnational policies, as well as qualitative data from key stakeholders, to gain a comprehensive understanding of how equity is translated from policy to practice in child health in the DRC.

## Conclusion

This study revealed substantial gaps in addressing equity in child health policy documents in the DRC. The policies lacked operational detail, measurable actions, and monitoring mechanisms to support implementation. These gaps may limit the effectiveness of child health programmes and hinder efforts to achieve equitable outcomes in the country. The findings of this study suggest that future efforts should not only aim to improve child health outcomes, but also enhance the adoption of equity considerations throughout policy development, implementation, and M&E. Moving from broad statements of intent to concrete and actionable equity commitments is essential for building a health system that guarantees every child receives care according to their needs and ensures that all children, regardless of background or circumstance, can achieve their full health potential.

## Data Availability

The data supporting the findings of this review are available from the corresponding author upon reasonable request.

## Abbreviations

CC: Core Concept
CMYPI: Comprehensive multi-year plan for immunization
LMICs: Low- and middle-income countries
MNCH: Maternal, newborn, and child health
M&E: Monitoring and evaluation
MoH: Ministry of Health
NHDP: National Health Development Plan
DRC: Democratic Republic of the Congo
RMNCAH-NUT: Reproductive, maternal, newborn, child, and adolescent health-nutrition
SDG: Sustainable Development Goal
UHC: Universal health coverage
UNICEF: United Nations Children’s Fund
WHO: World Health Organization
